# Clinical therapeutic effects of gastrodin in combination with betahistine on vertigo: A protocol for systematic review and meta-analysis

**DOI:** 10.1097/MD.0000000000023825

**Published:** 2021-03-12

**Authors:** Yu-Lin Qiao, Wen-Qiang Xiang, Fang Liu, Sheng Jin

**Affiliations:** a Department of Neurology, Hanyang Hospital of Wuhan; b Department of Neurology, the Fourth Hospital of Wuhan; c Department of Respiratory Medicine, Hubei NO.3 People's Hospital of Jianghan University; d Nephrology Department of Integrated Traditional Chinese and Western Medicine, Hubei NO.3 People's Hospital of Jianghan University, Wuhan, Hubei, China.

**Keywords:** vertigo, gastrodin, betahistine, effective, meta-analysis

## Abstract

**Background:**

Vertigo is a well-known presenting complaint common in the main care offices as well as departments. It is also regarded as a symptom of vestibular dysfunction and has been expressed as a feeling of motion, specifically rotational motion. As patients grow older, vertigo also becomes a commonly presenting complaint. The current study will carry out a widespread systematic review to estimate clinical therapeutic effects of gastrodin in combination with betahistine on vertigo.

**Methods:**

We will systematically search different databases, including PubMed, EMBASE, Web of Science, the Cochrane Library, Chinese BioMedical Literature Database (CBM), China National Knowledge Infrastructure Database (CNKI), and WanFang to collect the randomised controlled studies that evaluate the efficiency of gastrodin and betahistine in treating patients with vertigo from their inception to November 2020. However, only studies in English or Chinese will be included. Two authors will independently perform selection, data extraction, and assessment of risk of bias for the included papers. Accordingly, any disagreements between the independent authors will be addressed via discussion or by consulting a third author when needful. Additionally, we will use RevMan 5.3 software to perform the data synthesis.

**Results:**

The efficiency of gastrodin and betahistine in treating patients with vertigo will be systematically evaluated.

**Conclusions:**

The current study aims to stipulate more consistent substantiation to explore whether gastrodin combined with betahistine is more effective for the treatment of vertigo.

**Registration number:**

DOI 10.17605/OSF.IO/HQTZA (https://osf.io/hqtza/)

## 1 Introduction

Vertigo denotes a type of dizziness or the delusion of motion. Particularly, it is expressed as an observed delusion of motion of either self or environment in the absence actual physical movement.^[1,2]^ The most well-known causes of vertigo include peripheral or central vestibular (or ocular motor disorder), and rarely non-vestibular disorders or functional disorders.^[3]^ In essence, the main aetiologies of vertigo primarily originate from dysfunction of brainstem-cerebellar vestibular vertigo, ocular motor, or sensorimotor circuits. More importantly, cerebellar vertigo is a common term for this group of disorders that share similar symptoms of cerebellar dysfunction on clinical investigation of ocular motor, vestibular, or postural systems.^[4–6]^ Therefore, it is critical to distinguish vertigo from other types like imbalance and light-headedness. Accordingly, therapy for patients experiencing vertigo often consider a multimodal aspect and needs, including physical exercises for eye balance, stance, gait control, as well as pharmacological therapy.^[7]^ Still, these therapies have limited efficacy, and thus, are usually supplemented by some adverse reactions.^[8,9]^

Gastrodin is an effective monomeric component extracted from the traditional Chinese medicine, Gastrodia. It presents the functions of expanding cerebral blood vessels, improving brain cells tolerance to hypoxia, reducing cerebrovascular resistance, and increasing cerebral blood flow.^[10]^ Foregoing studies have illustrated that gastrodin can present an excellent clinical effect on vertigo due to various reasons.^[11]^ In recent years, there have been many clinical studies of gastrodin combined with betahistine in the treatment of vertigo. Until now, it is not clear whether this treatment plan can quickly and effectively improve the symptoms of patients or whether its safety is guaranteed. Thus, the current study seeks to perform a wide-ranging systematic review to explore clinical therapeutic effects of gastrodin in combination with betahistine on vertigo.

## 2 Methods

This protocol has been registered on the Open Science Framework (OSF) (https://osf.io) and the registration DOI number is 10.17605/OSF.IO/HQTZA. The current study will be conveyed as per the guidelines of Preferred Reporting Items for Systematic Reviews and Meta-Analyses Protocols (PRISMA-P) statement.

### 2.1 Inclusion criteria for study selection

Types of studies

All of the randomised controlled trials (RCTs) of gastrodin in combination with betahistine for vertigo will be included.

#### 2.1.1 Types of participants

In our study, we will include all participants who have met the diagnostic criteria f vertigo irrespective of their ages, races, or gender.

#### 2.1.2 Types of interventions

We will also include RCTs which involved gastrodin in combination with betahistine that can measure the therapeutic effect of vertigo as the experimental interventions. Gastrodin, betahistine, placebo, or no treatment groups will be included as the control interventions.

#### 2.1.3 Types of outcome measures

The major outcomes might include clinical efficacy, vertigo symptoms, and visual analogue scale. The minor outcomes will include adverse reactions.

#### 2.1.4 Search strategy

We will systematically search databases such as PubMed, EMBASE, Web of Science, the Cochrane Library, Chinese BioMedical Literature Database (CBM), China National Knowledge Infrastructure Database (CNKI), and WanFang to collect randomised controlled studies that evaluate the efficiency of gastrodin and betahistine to treat patients with vertigo from their inception to November 2020. However, we will only include papers in English or Chinese. To avoid losing any available literature that might meet the needs of the present study, we will search ClinicalTrials.gov (https://clinicaltrials.gov/), Google Scholar, and the reference lists of the included studies to identify any further studies. Search terms consist of intervention (gastrodin∗ OR betahistine∗) and disease (vertigo∗) and study methods (RCT∗ OR “randomised controlled trial” OR “randomised controlled trial” OR “random trials”).

### 2.2 Data collection and analysis

#### 2.2.1 Study selection

**Figure 1:**
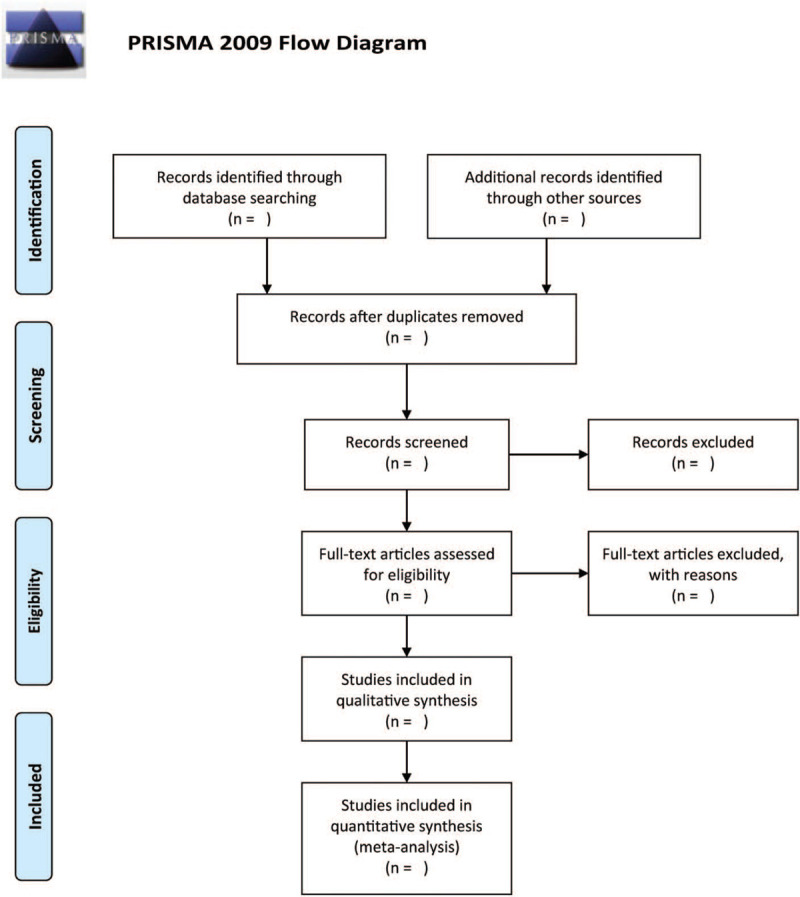
The research flowchart.

We will then utilize the Endnote X9 software to record and manage literature. Accordingly, 2 authors will independently study all the eligible papers for inclusion. They will scrutinize titles and abstracts of each of the articles and remove all unrelated studies. Also, 2 authors will independently review selected eligible studies on the basis of full-texts. Additionally, any disagreements between the authors will be addressed via discussion or by consulting a third author when needful. Figure 1 illustrates the flow diagram of study selection.

#### 2.2.2 Data extraction and management

Two authors will independently utilise a data collection form, which was piloted to be utilised on 1 study to examine both the outcome and characteristic data. The extracted data include:

1. participants: number of participants, mean age, gender, diagnostic criteria, inclusion, and exclusion criteria;
2. methods: study design, setting of the study, randomization method, start and end dates, and study location;
3. description of intervention and comparator: intervention medications, comparator medications, concomitant medications, duration of treatment;
4. outcomes: major and minor outcomes specified and collected, and time points reported.

Accordingly, any disagreements between the authors will be addressed via discussion or by consulting a third author when needful.

#### 2.2.3 Assessment of risk of bias

Furthermore, 2 authors will independently utilize literature quality evaluation items in Cochrane Reviewer's Handbook 5.1 published by the Cochrane Collaboration to assess the quality of the literature and then cross-check them accordingly.^[12]^ Any disagreements between the authors will be addressed via discussion or by consulting a third author when needful.

#### 2.2.4 Measurement of treatment effect

Regarding dichotomous outcomes, we will intend to utilize the collected data for calculation of the risk ratios (RR) as well as 95% confidence intervals (CI). For continuous outcomes, we will intend to use these data for calculating the mean difference (MD) or standardized mean difference (SMD) together with 95% CI.

#### 2.2.5 Assessment of heterogeneity

The *I*^2^ statistic and Chi^2^ test will also be utilized to examine statistical heterogeneity across studies. An *I*^2^ > 50% or *P* < .1, will depict that the data is significantly heterogeneous. The, we will apply the random-effects model; otherwise, we will utilize the fixed-effects model.

#### 2.2.6 Sensitivity analysis

We intend to undertake sensitivity analyses to evaluate our findings robustness when identifying sufficient data.

#### 2.2.7 Assessment of reporting biases

We will plan to undertake the funnel plots to explore potential publication bias when we include more than 10 studies.

### 2.3 Ethics and dissemination

As the study was a meta-analysis, ethical approval was not required.

## 3 Discussion

Gastrodin is a conventional Chinese medicine, mainly used as an external treatment method in clinical treatment. While gastrodins benefits for vertigo have been assessed, the clinical therapeutic effects of gastrodin has not been systematically examined. Thus, it is needful to carry out a meta-analysis to explore the clinical therapeutic effects of gastrodin in combination with betahistine on vertigo. The current study aims to summarise all evidence regarding published observational studies and provide evidence-based suggestions for vertigo treatment.

## Author contributions

**Data curation:** Yulin Qiao, Fang Liu, Sheng Jin.

**Formal analysis:** Yulin Qiao, Wenqiang Xiang.

**Funding acquisition:** Fang Liu, Sheng Jin.

**Investigation:** Wenqiang Xiang, Sheng Jin.

**Methodology:** Wenqiang Xiang.

**Project administration:** Wenqiang Xiang, Sheng Jin.

**Resources:** Fang Liu, Sheng Jin.

**Software:** Yulin Qiao.

**Validation:** Wenqiang Xiang, Fang Liu, Sheng Jin.

**Visualization:** Wenqiang Xiang.

**Writing – original draft:** Yulin Qiao, Fang Liu, Sheng Jin.

**Writing – review & editing:** Yulin Qiao, Fang Liu.
